# The relationship between the age of onset of musical training and rhythm synchronization performance: validation of sensitive period effects

**DOI:** 10.3389/fnins.2013.00227

**Published:** 2013-11-29

**Authors:** Jennifer A. Bailey, Virginia B. Penhune

**Affiliations:** Department of Psychology, Concordia UniversityMontreal, QC, Canada

**Keywords:** musical training, sensitive period, brain development, early training, working memory

## Abstract

A sensitive period associated with musical training has been proposed, suggesting the influence of musical training on the brain and behavior is strongest during the early years of childhood. Experiments from our laboratory have directly tested the sensitive period hypothesis for musical training by comparing musicians who began their training prior to age seven with those who began their training after age seven, while matching the two groups in terms of musical experience (Watanabe et al., 2007; Bailey and Penhune, 2010, 2012). Using this matching paradigm, the early-trained groups have demonstrated enhanced sensorimotor synchronization skills and associated differences in brain structure (Bailey et al., 2013; Steele et al., 2013). The current study takes a different approach to investigating the sensitive period hypothesis for musical training by examining a single large group of unmatched musicians (*N* = 77) and exploring the relationship between age of onset of musical training as a continuous variable and performance on the Rhythm Synchronization Task (RST), a previously used auditory-motor RST. Interestingly, age of onset was correlated with task performance for those who began training earlier, however, no such relationship was observed among those who began training in their later childhood years. In addition, years of formal training showed a similar pattern. However, individual working memory scores were predictive of task performance, regardless of age of onset of musical training. Overall, these results support the sensitive period hypothesis for musical training and suggest a non-linear relationship between age of onset of musical training and auditory-motor rhythm synchronization abilities, such that a relationship exists early in childhood but then plateaus later on in development, similar to maturational growth trajectories of brain regions implicated in playing music.

## Introduction

A sensitive period is a window in development when specific training or experience produces long-term changes in behavior and the brain, above and beyond those associated with that same experience at a different time during development (Knudsen, 2004; de Villers-Sidani and Merzenich, 2011). Sensitive periods have been proposed for the visual and auditory systems, as well as for language learning (for reviews see Hensch, 2005; Hooks and Chen, 2007; de Villers-Sidani and Merzenich, 2011). A sensitive period for musical training has also been proposed based on evidence that early-trained musicians demonstrate advantages over late-trained musicians for musical tasks such as rhythm synchronization and pitch identification, as well as differences in brain structure, particularly in motor regions (Takeuchi and Hulse, 1993; Schlaug et al., 1995; Amunts et al., 1997; Steele et al., 2013). Recent studies from our laboratory have shown that musicians who begin training before age seven perform better on auditory and visual Rhythm Synchronization Tasks (RSTs) even when groups are matched for years of experience, formal training and hours of current practice (Watanabe et al., 2007; Bailey and Penhune, 2010, 2012). More recently, we have also shown that early trained musicians have greater gray matter in the pre-motor cortex and greater white matter integrity in the corpus callosum (Bailey et al., 2013; Steele et al., 2013). These studies compared early- and late-trained musicians using age seven as the dividing point between the groups. Although chosen based on previous findings (i.e., Schlaug et al., 1995), it can be argued that such a cut-point is arbitrary. Furthermore, sensitive periods arise due to the interaction between specific experience and the maturational trajectories of implicated brain regions. Most of the maturational trajectories of the auditory and motor regions implicated in musical training follow non-linear growth curves (Gogtay et al., 2004; Lebel et al., 2008). Previous studies of second language proficiency have shown that the age at which people acquire a second language shows a non-linear relationship with performance, likely mirroring the maturational trajectories of the relevant language regions (Johnson and Newport, 1989; Flege et al., 1999). Therefore, the purpose of the current study was to examine auditory-motor synchronization performance in a large sample of musicians who began training across a range of ages. Using this data, we then tested to see whether age of start of musical training and task performance followed a linear or a non-linear relationship. In addition, we examined the contribution of other factors, including years of formal musical training and individual differences in auditory working memory.

Previous studies support the sensitive period hypothesis for musical training by reporting differences in brain structure or task performance between groups of early- and late-trained musicians. One of the first studies reported greater corpus callosum surface area among musicians compared to non-musicians and showed that these differences were greater for those who began their training before age seven (Schlaug et al., 1995). In addition, differences in the corticospinal tract between early- and late-trained musicians have been reported (Imfeld et al., 2009), as well as a negative correlation between age of start of musical training and intrasulcal length of the precentral gyrus among keyboardists (Amunts et al., 1997). These results support the idea of a sensitive period for musical training, however, these studies did not control for the confounding fact that those who begin earlier likely have had more musical experience at the time of testing than their late-trained counterparts. Work in our laboratory has controlled for this by using a matching paradigm where groups of early- (ET; < 7) and late-trained (LT; > 7) musicians are matched for years of total playing experience, as well as years of formal training and hours of weekly practice. Evidence using this paradigm has provided more direct support for a sensitive period for musical training, such that ET musicians have consistently outperformed LT musicians on both visual-motor (Watanabe et al., 2007) and auditory-motor synchronization tasks (Bailey and Penhune, 2010, 2012). More recently, differences observed in the corpus callosum and the pre-motor cortex between ET and LT musicians have been identified using the matching approach (Bailey et al., 2013; Steele et al., 2013). As described above, a sensitive period arises when specific experience interacts with a particular phase of brain maturation. Average anatomical maturational trajectories of gray matter and white matter in several regions of the brain follow non-linear growth curves, with peaks varying between ages 5 and 10 years old with continued, but more subtle change thereafter (Gogtay et al., 2004; Lebel et al., 2008). In other words, the maturation rates in these areas are not consistent across development and have ages at which maturation is greatest, as well as ages at which point maturation slows or plateaus. The evidence reporting the effects of musical training on brain structure is accumulating (for review see Jäncke, 2009; Wan and Schlaug, 2010), however, what remains to be investigated is whether this effect is of a linear nature or whether it mimics the maturational trajectories in the brain and is of a non-linear nature. Previous evidence cited compared groups of early- and late-trained musicians and these findings could be explained by either a linear or a non-linear effect. Generally speaking, it may be the case that the earlier an adult musician begins their training, the better they will perform on a musical rhythm task, suggesting a linear relationship between age of onset of musical training and task performance. However, it may also be that the age at which an adult musician begins their training provides an advantage when performing a musical rhythm task only up to a certain age, after which point, the age of training commencement offers little advantage, depicting a non-linear relationship between age of onset of musical training and task performance. Using a single, large sample of musicians with a wider distribution of age of onset and years of formal training provides a complementary approach to the previously used group comparisons in order to empirically examine the type of relationship between age of onset of musical training and task performance.

If a non-linear effect is present in the data, the predictive strength of age of onset of musical training for task performance may vary as a function of when musical training begins. More specifically, if a sensitive period for musical training does exist, the age at which an individual begins within this window during development (e.g., 4 years old) is likely to predict auditory-motor synchronization performance, however, if an individual begins training outside of this window, the age at which they begin (e.g., 16 years old) may predict their task performance to a much lesser degree. In some cases, with large enough samples, a non-linear effect may be concluded when a third-order function accounts for more variability in the data than a first-order function. Of more relevance to the question at hand, comparing pre- and post-correlation values before and after a break point associated with a sensitive period has also been used. A similar question has been investigated in the domains of second-language acquisition and cochlear implant research. Of these studies, the most relevant to the current data set and question is the work of Johnson and Newport (1989), who investigated the relationship between age of arrival in the United States and English proficiency among second-language learners. They reported that prior to puberty (< age 15), a significant correlation between age of arrival and proficiency measures was observed, but no such relationship was observed for individuals arriving after age 15. In other words, age of arrival was only predictive of English proficiency if it took place prior to age 15, supporting a non-linear relationship between age of arrival and English proficiency. Flege et al. (1999) reported further evidence for a non-linear relationship between age of arrival and language proficiency measures in second-language learners. They observed that simple correlations were present for certain ranges of age of arrival but that the correlation was not always consistent across the age range. One of the techniques they used to examine this was testing for discontinuities in their data by selecting specific ages as break points to divide the groups and then comparing the correlations in each group. The analyses in the current study have been largely based on the methods implemented in these studies in order to examine whether a linear or non-linear relationship exists between age of onset of musical training and auditory-motor synchronization performance. In addition to comparing pre- and post-correlations after break point ages, slopes were also compared. Given that these analyses were designed to be exploratory in nature, there were no precise predictions for the current data set.

While age of onset of musical training has typically been the variable of interest for our lab, years of formal training and individual working memory scores are additional characteristics that have shown a relationship with auditory-motor synchronization performance on the RST (RST; Bailey and Penhune, 2010, 2012). This task requires participants to tap in synchrony with a series of auditory rhythms of varying metrical complexity (Chen et al., 2008). Previous studies using ET and LT musicians have revealed that performance on the RST is related to brain structure, musical training and cognitive abilities. ET musicians have been better able to reproduce the temporal structure of the rhythms, with no group differences on standard measures of global cognitive function (Vocabulary and Matrix Reasoning), however, individual working memory scores (Digit Span and Letter-Number Sequencing) have correlated with RST performance (Bailey and Penhune, 2010, 2012). A regression analysis confirmed that, even after considering individual working memory scores, early training accounted for additional variance in RST performance. Similar to working memory, individual years of formal musical training also related to RST performance, even though the musician groups were matched on this variable (Bailey and Penhune, 2010, 2012). Taken together, these results indicate that RST performance is predicted by age at which musical training begins, the number of years of formal training and individual working memory abilities in musicians. Given that the sensitive period may result from an interaction between maturational trajectories of brain regions and experience, it stands to reason that the predictive value of all three of these variables for RST performance may change, depending on when musical training began. One could expect that years of formal training would predict task performance more strongly among those who received years of training in their early childhood years compared to those who received their training in later years. Similarly, the predictive value of working memory scores for performance on the RST might change as a function of when musical training occurred during development. Using an unmatched large single sample of musicians, with a wider distribution of age of start and years of formal training, we have taken the opportunity to investigate these questions to further our understanding of a sensitive period for musical training.

In summary, the current study will explore the nature of the relationship between age of onset of musical training and performance on the RST in a large sample of musicians by first considering a linear correlation model, followed by breakpoint analyses comparing correlation values to determine if the relationship between age of onset and RST performance changes across development, similar to Johnson and Newport's approach (1989) and Flege et al. (1999) exploration of discontinuities in their data sets. Finally, changes in the predictive value of years of formal training and individual working memory scores on RST performance based on when musical training began will be explored. This is the first study to examine the nature of the relationship underlying the sensitive period effect for musical training using a single sample of musicians with a wide range in age of onset of musical training as well as length of formal training.

## Materials and methods

### Participants

The current study uses a sample of 77 musicians between the ages of 18 and 37 (*M* = 24.91, *SD* = 4.97). This sample includes musicians previously tested in studies comparing early- and late-trained musicians using a matched samples design (Bailey and Penhune, 2010, 2012). For this study we tested additional musicians to cover a broader range of ages of start (3–17). The musical training and experience of each participant was determined through a Musical Experience Questionnaire (MEQ) that was developed within our laboratory (Bailey and Penhune, 2010, 2012). The MEQ quantifies the amount of instrumental and vocal training a musician has received, age of onset of this training, number of years of formal lessons and the amount of time dedicated to practicing on a weekly basis at the time of testing. Musicians had a range of musical experience (Table 1). All participants were neurologically healthy and were screened for significant head injuries, history of neurological disease or medication that could affect task performance. All participants gave informed consent and the Concordia University Research Ethics Committee had approved the protocol.

**Table 1:** **Musical demographics**.

	**Age of onset (years)**	**Formal training (years)**	**Playing experience (years)**	**Current practice (hours)**
Mean	8.43 (±3.57)	10.09 (±4.79)	15.99 (±4.32)	17.28 (±11.12)
Range	3–17	0–20	7–25	0–56

Standard deviations are in brackets.

### Tasks

**Figure 1:**
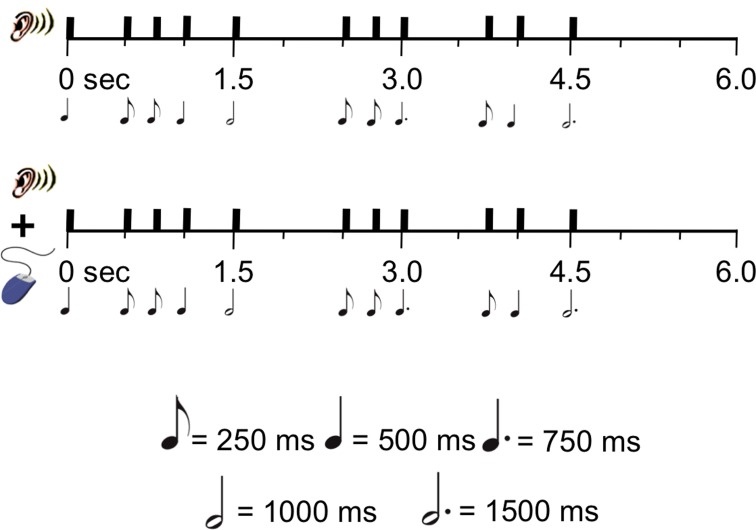
**An illustration of the Rhythm Synchronization Task (RST)**.

Participants performed the RST (Figure 1), which was previously used in Bailey and Penhune (2010, 2012) and is a variant of the task used in Chen et al. (2008). In this task, participants are required to listen to and then tap in synchrony with a series of auditory rhythms of varying metrical complexity. The task consists of six woodblock rhythms varying in metrical structure and difficulty. Each rhythm lasts 6 s and is made up of 11 woodblock notes. Each rhythm contains five eighth notes (250 ms), three quarter notes (500 ms), one dotted quarter note (750 ms), one half note (1000 ms) and one dotted half note (1500 ms). Each trial has two parts: during the first part, participants listen to the rhythm without responding, and during the second part they listen and tap in synchrony using the computer mouse. Key press responses are recorded by the computer and used to score the data as described below. For a more detailed description of the RST, please see Bailey and Penhune (2010, 2012).

Participants completed the Digit Span and Letter-Number Sequencing subtests from the Wechsler Adult Intelligence Scale-III (WAIS) and the Vocabulary and Matrix Reasoning subtests from the Wechsler Abbreviated Scale of Intelligence (WASI; Wechsler, 1997, 1999). Digit Span requires individuals to recall strings of numbers and Letter-Number Sequencing requires individuals to recall and mentally manipulate strings of letters and numbers. Both of these subtests tap into working memory abilities, however, Letter-Number Sequencing imposes a heavier load on working memory, while Digit Span consists of a rote auditory memory recall section in addition to a mental manipulation section. Vocabulary assesses an individual's ability to orally define words and Matrix Reasoning assesses non-verbal reasoning and visual pattern recognition abilities. Both of these subtests are highly correlated with global IQ, yet are thought to represent different aspects of intelligence (Wechsler, 1999).

### Procedure

All participants followed the same procedure for data collection. Participants first completed one block of the RST followed by the Digit Span test. They then performed the second block of the RST, followed by Vocabulary, Letter-Number Sequencing and finally, Matrix Reasoning.

### Measures

**Figure 2:**
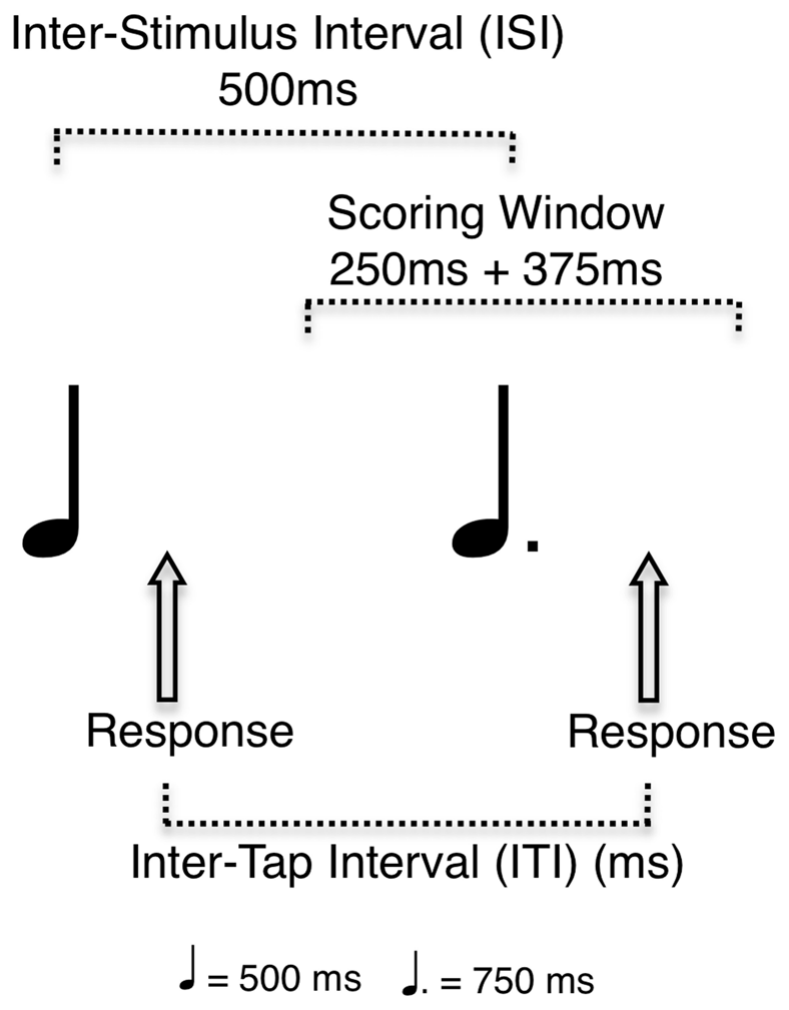
**An illustration of the scoring practices used to determine performance on the Rhythm Synchronization Task (RST)**.

Information about musical training and experience from the MEQ was quantified for each participant to produce measures of years of experience, years of formal training and hours of weekly practice. Cognitive subtest results were scored according to standard procedure. A composite score for each participant's working memory abilities was created using their Letter-Number Sequencing and Digit Span scores and was used as the Working Memory variable. Performance on the RST was measured using three dependent variables: percent correct (PC), asynchrony (ASYN), and inter-tap-interval (ITI) deviation. A tap was considered correct if it was made within half of the onset-to-onset interval before or after a woodblock note (Figure 2). ASYN was defined as the absolute value of temporal difference between the onset of each woodblock note and the associated mouse key press. ITI deviation was calculated by dividing the interval between each pair of the participant's taps by the interval between each corresponding pair of woodblock notes in the rhythms and subtracting this ratio from a value of one. This measure evaluates the extent of deviation of the participant's tap interval from the actual interval between each pair of woodblock notes and is indicative of how well participants reproduce the temporal structure of the rhythms.

### Data analysis

In order to replicate findings from Bailey and Penhune (2010, 2012) that age of onset of musical training, individual working memory scores and amount of formal training contribute to RST performance in this larger and unmatched sample, one-tailed Pearson correlation analyses were conducted between the variables: ASYN, ITI Deviation, Age of Onset, Working Memory, and Formal Training. PC was not analyzed because it is a global measure of task performance and has not previously revealed group differences between early-trained musicians and late-trained musicians, nor is it informant about the exact timing of participant taps (Chen et al., 2008; Bailey and Penhune, 2010, 2012).

To test for evidence of break points in the data, the musicians were split using four different age of onset values. ET and LT groups were defined by using ages 6–9 (ET ≤ 6, *n* = 30, LT > 6, *n* = 47; ET ≤ 7, *n* = 38, LT > 7, *n* = 39; ET ≤ 8, *n* = 45, LT > 8, *n* = 32; ET ≤ 9, *n* = 50, LT > 9, *n* = 27). Correlation analyses were conducted between age of onset and RST performance for each of the ET and LT groups. Correlation coefficients were compared in each condition by calculating a *z*-test statistic according to the method designed by Fisher and slopes were calculated using regression models and compared using *t*-test analyses. Subsequently, formal training and working memory variables were correlated with task performance in the ET and LT groups separately using the strongest break-point age of onset value. These analyses were conducted to investigate differences in predictive strength of task correlates as a function of age of onset of musical training.

## Results

**Figure 3:**
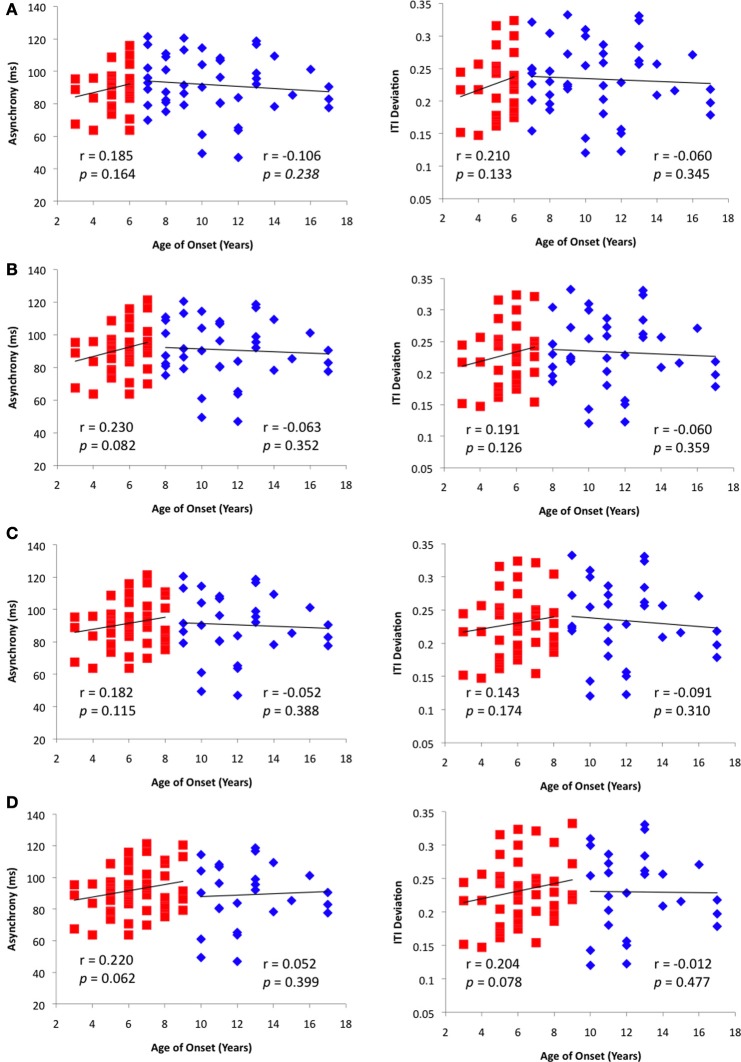
**Results from the breakpoint analyses conducted using ages (A) 6, (B) 7, (C) 8, and (D) 9 as different break points.** In each panel, age of onset was correlated with Rhythm Synchronization Task (RST) performance as measured by Asynchrony (ms) and Inter-tap Interval Deviation.

Age of onset of musical training did not significantly correlate with task performance measures using a linear correlation model with all musicians (Table 2), supporting the possibility of a non-linear relationship between age of onset of musical training and RST performance. In fact, using the four different break points in age of onset (i.e., ages 6–9) to split the musicians into ET and LT groups yielded results suggesting a non-linear relationship between age of onset of musical training and RST performance. All four break point conditions resulted in differential correlations between groups, with the ET group showing a positive correlation between age of onset and task performance (ASYN and ITI Deviation) and the LT group showing no correlation between age of onset and task performance. Of the four different conditions, when age 9 was used to divide the groups, the correlations between age of onset and task performance reached trend-level in the ET group (Figure 3D) and provide the strongest evidence supporting a non-linear relationship. However, the results from the Fisher transformation tests and slope comparison analyses suggest that the relationship between age of onset and task performance is most different when age 7 was used to divide the groups. The correlation results in each of the break point conditions are illustrated in Figure 3 and the results from the Fisher transformation tests and slope comparisons can be found in Tables 3, 4. Taken together, these results provide evidence that age of onset differentially predicts performance when training begins earlier as compared to later in development, however, there may exist a subtle change in effect around age 7 through 9. These results do not support a discrete cut point, but instead an age-range after which the predictive value of age of start of musical training plateaus.

**Table 2:** **Pearson correlation analyses of musical demographics, working memory scores, and RST Performance**.

**RST performance measures**	**Age of onset (years)**	**Formal training (years)**	**Working memory**
Asynchrony (ASYN)	−0.001	−0.118	−0.396^**^
Inter-tap interval (ITI) deviation	0.032	−0.224^*^	−0.464^**^

A composite score for working memory was created from raw scores on the digit span and letter-number sequencing cognitive subtests.

* p < 0.05.

** p < 0.001.

**Table 3:** **Comparison of Pearson correlation coefficients of task performance and age of onset between early- and late-trained musicians in each age of onset break point condition**.

**Age of onset cut-off (years)**	**Early-trained (ET) correlation coefficient (ASYN/ITI)**	**Late-trained (LT) correlation coefficient (ASYN/ITI)**	**Fisher's transformation z-value (ASYN/ITI)**
ET ≤ 6 > LT	0.185/0.210	−0.106/−0.060	1.2/1.12
ET ≤ 7 > LT	0.230/0.191	−0.063/−0.060	1.25^†^/1.07
ET ≤ 8 > LT	0.182/0.143	−0.052/0.091	0.98/0.22
ET ≤ 9 > LT	0.220/0.204	0.052/−0.012	0.68/0.87

† p = 0.1.

**Table 4:** **Comparison of slope values between early- and late-trained musicians in each age of onset break point condition**.

**Age of onset cut-off (years)**	**Early-trained (ET) slope (ASYN/ITI)**	**Late-trained (LT) slope (ASYN/ITI)**	***t*-value (ASYN/ITI)**
ET ≤ 6 > LT	2.653 (2.659)/0.01 (0.009)	−0.659 (0.918)/−0.001 (0.003)	1.177/1.159
ET ≤ 7 > LT	2.893 (2.040)/0.008 (0.006)	−0.432 (1.125)/−0.001 (0.003)	1.427^*^/1.342^*^
ET ≤ 8 > LT	1.869 (1.537)/0.005 (0.005)	−0.418 (1.454)/−0.002 (0.004)	1.089/1.093
ET ≤ 9 > LT	1.984 (1.269)/0.006 (0.004)	0.455 (1.755)/0.000 (0.005)	0.706/0.937

Standard error values of unstandardized b coefficients (i.e., slope values) are in brackets.

* p < 0.1.

**Figure 4:**
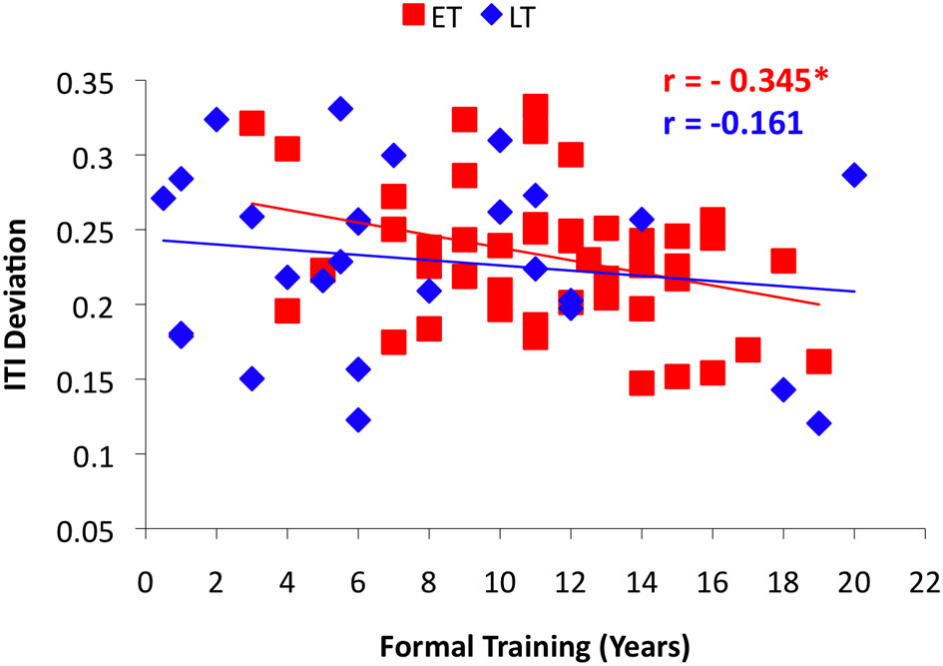
**Results from the correlation analyses between performance on the Rhythm Synchronization Task (RST) and years of formal training in each musician group using age 9 as the break point**.

**Figure 5:**
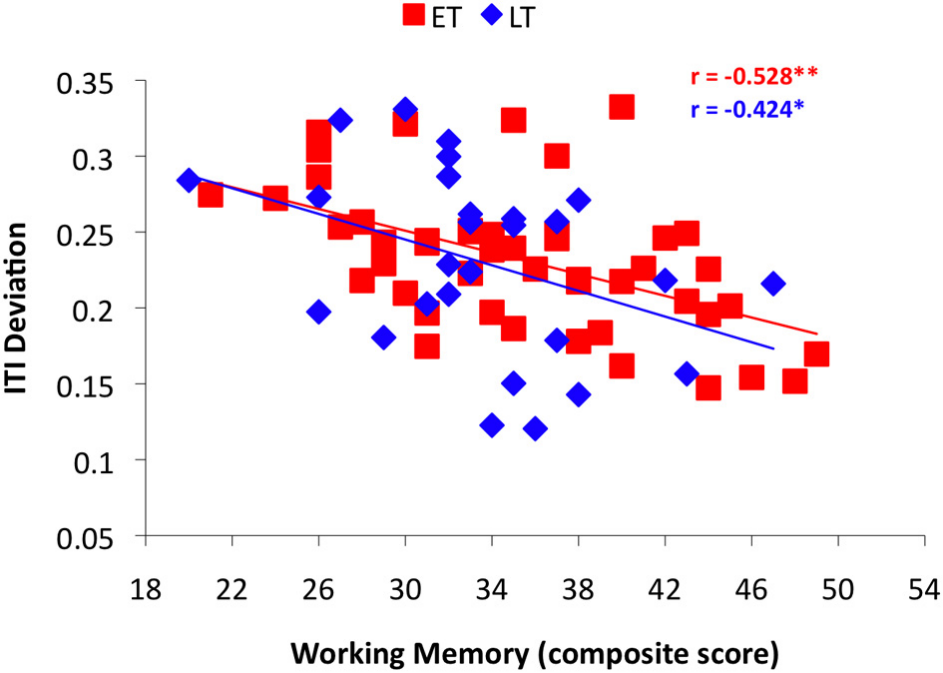
**Results from the correlation analyses between performance on the Rhythm Synchronization Task (RST) and working memory in each musician group using age 9 as the break point**.

**Figure 6:**
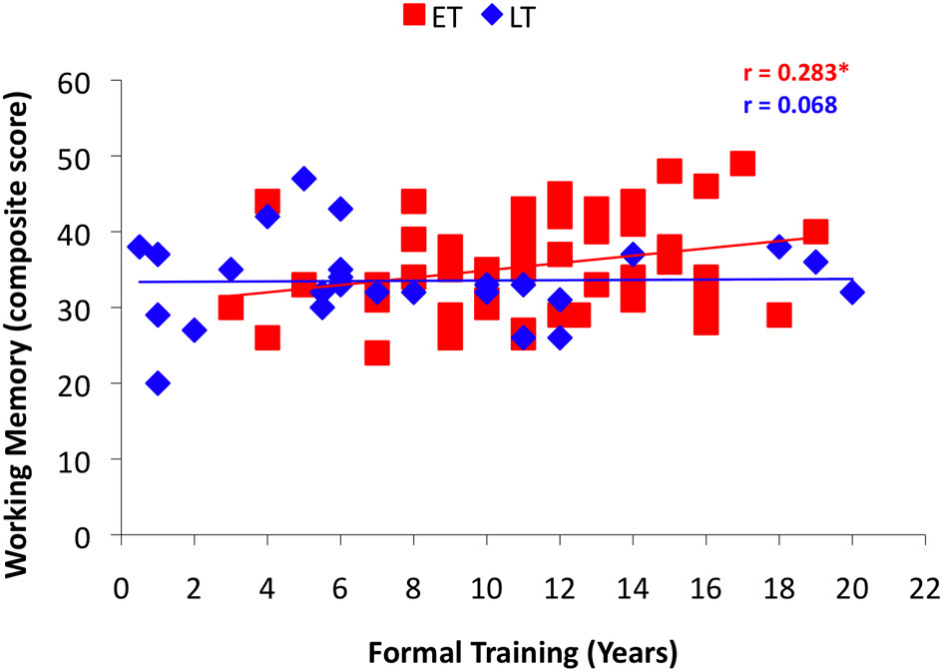
**Results from the correlation analyses between working memory and years of formal training in each musician group using age 9 as the break point**.

Linear correlation analyses across all musicians revealed a significant relationship between ITI Deviation and both working memory and formal training (Table 2), similar to previous findings. To investigate whether the predictive strength of task correlates differed based on age of start of training, years of formal training and working memory were examined in each musician group, using age 9 (ET ≤ 9, LT > 9) as the break point in the age of onset variable. A significant correlation between formal training and task performance (ITI Deviation) was observed for musicians who began training at age 9 or younger (Figure 4; *r* = −0.345, *p* < 0.01), however, this relationship was not significant among musicians who began training later (Figure 4; *r* = −0.161, *p* > 0.05). Working memory correlated with task performance in both groups (Figure 5). Finally, Figure 6 illustrates a significant relationship between formal training and working memory among those who began training earlier but not among those who began their training later. It should be noted that similar patterns for task correlates were observed when age 7 was used as the break point to divide the groups into ET and LT musicians.

## Discussion

The results from this study add to the growing body of evidence supporting a sensitive period for musical training. However, these findings are the first to examine whether a linear or non-linear relationship underlies the sensitive period effect for musical training. It is difficult to determine convincing evidence to support a sensitive period associated with musical training due to the effects of additional variables. The current evidence does not point to a single specific age cut-off, but instead to a more subtle age range when the effect of age of start of training decreases or plateaus. The simple correlation break point analyses suggest that age of onset predicts rhythm synchronization performance if musicians begin training at or prior to age 9, but not afterwards. The results from the Fisher's z-transformation analyses and slope comparisons suggest that the relationship between age of onset and task performance differs the most when age 7 is used to divide the groups. Examining task correlates using age 9 to split musicians into Early-Trained and Late-Trained groups revealed that performance on the RST, as assessed by ITI Deviation, correlated with years of formal training only in the Early-Trained group. Working memory scores correlated with ITI Deviation in both groups, however, this correlation was stronger among those who began their training prior to or at age 9. It is important to note that when task correlates were compared between groups using age 7 as a dividing age, the same pattern of findings was observed. Overall, these results suggest that effects associated with age of onset or amount of formal training on the RST are stronger earlier in development, with a change occurring between ages 7 and 9 and may plateau thereafter. While these results are consistent with previous findings reporting a group difference between early- and late-trained musicians, they introduce the idea of a non-linear relationship between aspects of musical training (e.g., age of start and years of formal lessons) and auditory-motor synchronization skills across development, mirroring the maturational growth trajectories of the brain regions implicated in playing music.

Previous studies from our laboratory have investigated a sensitive period for musical training by comparing groups of early- and late-trained musicians (before and after age seven) who were matched for years of experience in an effort to isolate the effects of age of onset (Watanabe et al., 2007; Bailey and Penhune, 2010, 2012; Bailey et al., 2013; Steele et al., 2013). In contrast, the current study was designed to determine the nature of the relationship between age of onset of training and auditory-motor rhythm synchronization abilities in a single sample of musicians. The results from the correlation analyses support the hypothesis that the relationship between age of onset and task performance is not linear across development. These results are supported by previous research examining sensitive periods in the language and auditory domains showing that age of acquisition of a second language or a cochlear implant and skill development is not a linear relationship across development, but instead reveals evidence for sensitive periods in development when this relationship is strongest (Johnson and Newport, 1989; Flege et al., 1999; Svirsky et al., 2004; Harrison et al., 2005). Furthermore, a non-linear relationship between age of onset and auditory-motor synchronization mirrors the maturational trajectories of the brain regions that comprise the auditory-motor neural network (Gogtay et al., 2004; Lebel et al., 2008). Maturational trajectories for gray and white matter in several brain regions follow a non-linear growth curve, with peaks varying between ages 5 and 10, depending on the region (Gogtay et al., 2004; Lebel et al., 2008). The primary motor cortices are among the first to mature (approximate peak at or prior to age 5; Gogtay et al., 2004), while the pre-motor cortex has a more protracted development (approximate peak at age 8.5; Gogtay et al., 2004). The posterior midbody of the corpus callosum connects the sensorimotor cortices of the two hemispheres (Hofer and Frahm, 2006; Chao et al., 2009) and this region undergoes significant developmental changes between the ages of 6 and 8 (Westerhausen et al., 2011). Interestingly, our previous studies using the matching paradigm reported differences between ET and LT musicians in the pre-motor cortex and the posterior midbody of the corpus callosum (Bailey et al., 2013; Steele et al., 2013).

Given that sensitive periods likely arise due to an interaction between maturational processes and experience, the current behavioral findings support and mimic the non-linear growth curves that have been observed during brain structure development. Previous studies provided evidence for a sensitive period around age seven, even though these studies were not designed to predict a specific age break point. The current results are not contradictory to these previous findings, but offer empirical evidence that ages 7 through 9 may be a period where a non-linear break in the relationship between age of start of musical training and auditory-motor synchronization skills takes place. The present findings suggest that the age of onset of musical training predicts auditory-motor synchronization abilities, if that training happens prior to a certain age range (7–9) but this effect stabilizes later in development, when brain networks are more mature and therefore less influenced by experience or training. Given the current exploratory nature of these analyses, it would be necessary to replicate these findings in a larger sample with more equal representation across age of onset as well as more accurate measures of age of onset (e.g., months), thus allowing the use of more stringent criteria in order to conclude the presence of a non-linear relationship between age of onset of musical training and auditory-motor synchronization abilities in adulthood.

A secondary, but related, finding from the current study is that formal training relates to RST performance only in early starters. Given the strong correlation with age of onset of musical training (*r* = −0.534), it is not surprising that formal training shows a similar non-linear effect on RST performance. It may be that music lessons during the earlier years have a stronger influence on training auditory-motor synchronization skills that are implicated in the RST than music lessons during the later years. Alternately, there are potential differences in the type of formal instruction received in early childhood compared to during the later years. Musical training programs beginning before children are able to read focus on learning by listening to and reproducing music from an auditory model. These skills may be particularly relevant for training auditory-motor synchronization.

Unlike formal training, individual differences in working memory abilities were similarly related to RST performance across both musician groups. In addition, working memory scores were not significantly related to age of onset of musical training overall (*r* = −0.116, *p* > 0.1). This provides a good reminder that individual working memory abilities are important for RST performance, but do not seem to be related to age of onset of training. Furthermore, a relationship between individual working memory abilities and RST performance was also observed among a group of non-musicians (Bailey and Penhune, 2010, 2012), supporting the results that this relationship is unaffected by age of start of musical training.

Overall, the current study provides additional evidence for the sensitive period hypothesis for musical training and offers a more nuanced view of the relationship between age of onset of musical training and auditory-motor synchronization abilities. These results suggest the presence of a non-linear relationship between age of onset of musical training and auditory-motor synchronization, such that the age at which training begins is related to auditory-motor synchronization abilities in adults, if that training begins in early childhood. This idea of a non-linear relationship is mirrored by growth trajectories of brains regions in the auditory-motor neural network and suggests that brain plasticity may decrease across development.

### Conflict of interest statement

The authors declare that the research was conducted in the absence of any commercial or financial relationships that could be construed as a potential conflict of interest.
